# In Utero Exposure to Maternal COVID-19 and Offspring Neurodevelopment Through Age 24 Months

**DOI:** 10.1001/jamanetworkopen.2024.39792

**Published:** 2024-10-16

**Authors:** Eleni G. Jaswa, Heather G. Huddleston, Karla J. Lindquist, Alan H. B. Wu, Somer L. Bishop, Young-Shin Kim, Amy Kaing, Mary Prahl, Stephanie L. Gaw, Jamie Corley, Elena Hoskin, Yoon Jae Cho, Elizabeth E. Rogers, Marcelle I. Cedars

**Affiliations:** 1 Division of Reproductive Endocrinology and Infertility, Department of Obstetrics, Gynecology and Reproductive Sciences, University of California, San Francisco; 2 Department of Epidemiology and Biostatistics, University of California, San Francisco; 3 Division of Clinical Chemistry, Department of Laboratory Medicine, University of California, San Francisco; 4 Department of Psychiatry, University of California, San Francisco; 5 Division of Pediatric Infectious Disease and Global Health, Department of Pediatrics, University of California, San Francisco; 6 Division of Maternal Fetal Medicine, Department of Obstetrics, Gynecology and Reproductive Sciences, University of California, San Francisco; 7 Division of Neonatology, Department of Pediatrics, University of California, San Francisco

## Abstract

**Importance:**

In utero exposure to maternal infections has been associated with abnormal neurodevelopment among offspring. The emergence of a new, now endemic infection (SARS-CoV-2) warrants investigating developmental implications for exposed offspring.

**Objective:**

To assess whether in utero exposure to maternal COVID-19 is associated with abnormal neurodevelopmental scores among children ages 12, 18, and 24 months.

**Design, Setting, and Participants:**

Data were ascertained from the ASPIRE (Assessing the Safety of Pregnancy in the Coronavirus Pandemic) trial, a prospective cohort of pregnant individuals aged 18 years or older who were enrolled before 10 weeks’ gestation and their children. Individuals were recruited online from May 14, 2020, to August 23, 2021, using the Society for Assisted Reproductive Technology and BabyCenter, an online media platform. Participants from all 50 states and Puerto Rico completed activities remotely.

**Exposure:**

In utero exposure to COVID-19.

**Main Outcomes and Measures:**

Birth mothers completed the Ages & Stages Questionnaires, Third Edition, a validated screening tool for developmental delays, at 12, 18, and 24 months’ post partum. A score below the cutoff in any domain (communication, gross motor, fine motor, problem-solving, and social skills) was considered an abnormal developmental screen (scores range from 0 to 60 in each domain, with higher scores indicating less risk for neurodevelopmental delay).

**Results:**

The cohort included 2003 pregnant individuals (mean [SD] age, 33.3 [4.2] years) enrolled before 10 weeks’ gestation and who completed study activities; 1750 (87.4%) had earned a college degree. Neurodevelopmental outcomes were available for 1757 children at age 12 months, 1522 at age 18 months, and 1523 at age 24 months. The prevalence of abnormal screens for exposed vs unexposed offspring at age 12 months was 64 of 198 (32.3%) vs 458 of 1559 (29.4%); at age 18 months, 36 of 161 (22.4%) vs 279 of 1361 (20.5%); and at age 24 months, 29 of 151 (19.2%) vs 230 of 1372 (16.8%). In an adjusted mixed-effects logistics regression model, no difference in risk of abnormal neurodevelopmental screens was observed at age 12 months (adjusted risk ratio [ARR], 1.07 [95% CI, 0.85-1.34]), age 18 months (ARR, 1.15 [95% CI, 0.84-1.57]), or age 24 months (ARR, 1.01 [95% CI, 0.69-1.48]). Supplemental analyses did not identify differential risk based on trimester of infection, presence vs absence of fever, or breakthrough infection following vaccination vs primary infection.

**Conclusions and Relevance:**

In this cohort study of pregnant individuals and offspring, exposure to maternal COVID-19 was not associated with abnormal neurodevelopmental screening results through 24 months’ post partum. Continued study of diverse groups of children is needed because, among other factors, evidence suggests sensitivity of the developing fetal brain to maternal immune activation.

## Key Points

**Question:**

Is exposure to maternal SARS-CoV-2 infection in utero associated with increased risk for neurodevelopmental impairment in early childhood up to age 24 months?

**Findings:**

In this cohort study of 2003 pregnant individuals and their children, the adjusted prevalence of abnormal scores on the Ages & Stages Questionnaires, Third Edition of children through age 24 months did not differ between offspring exposed and unexposed to maternal SARS-CoV-2 infection in utero.

**Meaning:**

These findings suggest that individuals infected with SARS-CoV-2 during pregnancy can be reassured that there is no association with abnormal neurodevelopmental scores in children through age 24 months.

## Teaser

This cohort study examines whether in utero exposure to maternal SARS-CoV-2 infection is associated with risk of neurodevelopmental impairment in children through age 24 months.

## Introduction

With the crisis phase of the COVID-19 pandemic over, the focus of medical research pivots to the potential ripple effects of the global outbreak. Unanswered questions endure for many vulnerable populations. One such population includes the cohort of children generated from pregnancies during the pandemic.

The in utero environment and early childhood mark a time of unparalleled, exponential growth and development. In the nature or nurture debate, scientific consensus is that nurture begins in utero^1^ and that in this unique period of vulnerability, in utero exposures may confer lifelong health consequences.^2,3^ In particular, in utero exposure to maternal viral infections has been associated with adverse neurodevelopment in offspring. Following the 1957 influenza pandemic^4,5^ and 1964 rubella pandemic,^6,7^ for example, increased rates of schizophrenia and autism spectrum disorder were observed. Animal data^8,9,10,11^ augment epidemiologic findings, offering mechanistic insights into potential neurologic risk pathways.

Notably, neurodevelopmental risk may exist even without direct vertical transmission of the infectious agent to the fetus. Instead, the destructive mechanisms are associated with maternal immune activation,^12,13,14^ which subsequently may influence key placental and fetal immune and inflammatory signaling pathways.^13^ Except for rare cases,^15,16,17^ the SARS-CoV-2 infection does not appear to cross the placenta, in which it could infect the fetus; however, maternal COVID-19 illness is, in many cases, associated with a significant inflammatory response with a potential impact on the in utero environment and therefore offspring development.^18^

Whether COVID-19 illness during pregnancy affects offspring neurodevelopment is of continued global relevance, with early studies yielding mixed results. Most^19,20,21^ but not all^22^ studies have found no association between in utero exposure to SARS-CoV-2 infection and alterations in scores on the Ages & Stages Questionnaires, Third Edition (ASQ-3),^23^ a widely used measure of risk for developmental delay. These null findings were echoed in a cohort study of infants using a telehealth developmental assessment tool.^24^ However, many studies have been limited by small sample sizes and short follow-up durations, primarily less than 1 year. Conversely, 2 retrospective electronic medical record billing code studies sparked concern about increased susceptibility to neurodevelopmental diagnoses, specifically among male offspring exposed to maternal COVID-19.^25,26^ In the setting of discordant results, the objective of our study was to investigate whether in utero exposure to maternal COVID-19 was associated with abnormal neurodevelopmental screening results measured by the ASQ-3 among children up to 24 months old in a large prospective cohort.

## Methods

### Study Design and Participants

This cohort study included data from the ASPIRE (Assessing the Safety of Pregnancy in the Coronavirus Pandemic) trial, a nationwide prospective cohort of pregnant individuals and offspring, which launched in April 2020. Participants who were aged 18 years or older and were newly pregnant (before 10 weeks’ gestation) were recruited online via partnerships with the Society for Assisted Reproductive Technology and BabyCenter, an online media platform with 32 million monthly users. Recruitment took place from May 14, 2020, to August 23, 2021. The University of California, San Francisco Institutional Review Board granted study approval, and participants provided written informed consent. The study followed the Strengthening the Reporting of Observational Studies in Epidemiology (STROBE) reporting guideline.^27^

Adult participants were followed through 24 months’ post partum. Study activities occurred remotely, including frequent online questionnaires and self-collection of timed dried blood spot (DBS) cards throughout pregnancy. Surveillance DBS cards were requested weekly through the first trimester and monthly through the second and third trimesters to generate the biobank.

We restricted our analysis to those who had completed the baseline questionnaire, at least 1 self-reported COVID-19 test or surveillance DBS, and the ASQ-3 at 12, 18, and/or 24 months’ post partum. Additional details regarding design, recruitment, and participants have been previously published.^28^

### Outcome

The primary outcome was an abnormal score on the ASQ-3,^23^ defined as scoring below the predefined threshold on any of 5 developmental domains: communication, gross motor, fine motor, problem-solving, and social skills. An abnormal ASQ-3 screening score is interpreted as indicating potentially increased risk for neurodevelopmental delay over a validated and age-normed baseline.

Age-specific versions of the ASQ-3 were completed at 12, 18, and 24 months’ post partum. The questionnaire’s 30 items assess the frequency with which a child performs developmentally appropriate milestones. Scores range from 0 to 60 in each domain, with higher scores indicating less risk for neurodevelopmental delay. Sensitivity and specificity are self-reported by the ASQ-3 as 86% and 85%, respectively.^29^ Additional details and sample questions are publicly available.^29^

### Exposure and Covariates

The primary exposure was maternal SARS-CoV-2 infection during pregnancy. This was ascertained by either self-report or DBS cards collected during pregnancy. The DBS cards collected throughout pregnancy were assayed for the SARS-CoV-2 spike protein immunoglobulin G using a published extraction procedure^30^ and pooled approach.^31^ A serologic approach was selected at study launch prior to the widespread availability of commercial antigen testing and in the context of the relative stability of antibodies on DBS cards, allowing for study activities to be compatible with shelter-in-place and other societal restrictions. Frequent surveillance of blood spots was intended to augment the self-report infection data, specifically in assessing for asymptomatic infection known early in the pandemic to constitute a meaningful proportion of COVID-19 cases in pregnancy.^32^

A threshold of 50 relative fluorescence units indicated a positive immunoglobulin G serology. In a feasibility study with a separate population of 25 individuals, we demonstrated 100% concordance between quantitative results for DBS vs whole blood results (internal data). A DBS card was considered positive for SARS-CoV-2 infection after excluding antecedent vaccination against COVID-19. Participants with neither self-reported SARS-CoV-2 infection during pregnancy nor a positive DBS card throughout pregnancy were considered unexposed.

We selected covariates a priori based on subject matter expertise. Potential confounders included maternal age, self-reported race and ethnicity, educational level, household income, maternal generalized anxiety symptoms at baseline (based on a Generalized Anxiety Disorder 7 score >4,^33^ in which scores range from 0 to 21 points, with higher scores indicating a severe level of anxiety), maternal depression symptoms at baseline (based on a Patient Health Questionnaire score >4,^34^ in which scores range from 0 to 27, with higher scores indicating the presence of severe depressive symptoms), and a continuous temporal measure marking the enrollment date relative to vaccine availability. Race and ethnicity categories included Asian, Black, Hispanic, White, and multiracial (including any combination of Asian, Black, Native American, Pacific Islander, or White) or other (including those who identified with groups not explicitly listed). Race and ethnicity were self-reported and included in the study to assess their association with health outcomes and to clarify the generalizability of the findings.

Vaccination was not included in the confounder set, as most of us have previously demonstrated no association with child neurodevelopment.^28^ Infant sex was considered as a potential effect modifier, and preterm birth was considered as a possible mediator. Trimester of infection, breakthrough infection (defined as a SARS-CoV-2 infection following vaccination compared with a primary infection not preceded by vaccination), and COVID-19–related fever (>38 °C) were subsequently considered in supplemental analyses to investigate further potential modulators of the association between primary exposure and outcome.

### Statistical Analysis

We used mixed-effects logistic regression models to investigate the association between prenatal infection and a high-risk screen for neurodevelopmental delay at 12, 18, and 24 months’ post partum. Fixed-effects with robust SEs were used for all covariates and included the infection status (the primary exposure), the month of the ASQ-3 screening, and an interaction between these 2 components. Models included random intercepts to account for within-participant correlation between ASQ-3 scores. An unstructured correlation matrix was used for random effects. Effect sizes for the association between all covariates and the primary outcome (abnormal ASQ-3 score) were expressed as relative risk ratios (RRs). The risk of a positive screen for neurodevelopmental delay at each time point, by infection status, was calculated using the time-exposure interaction term and marginal estimated probabilities.

Our primary analyses included a series of 3 models. We used model 1 results to calculate unadjusted RRs for the association between prenatal infection and an abnormal ASQ-3 score, including the interaction term between infection and month of outcome measurement. Model 2 subsequently added adjustment for the aforementioned confounder set. Finally, given the lack of evidence for effect modification by infant sex or mediation by preterm birth but with strong unadjusted associations between these covariates and the primary outcome, these 2 variables were added to model 3 as independent variables.

We conducted supplemental analyses to clarify our findings further and to address ensuing questions. To evaluate whether the timing of infection during gestation may have influenced the risk of abnormal neurodevelopment, we compared adjusted RRs (ARRs) by trimester of maternal infection. We evaluated whether developmental risk differed by febrile vs nonfebrile infection and whether primary infection without a previous vaccination conferred a different risk than a breakthrough infection following vaccination. We also conducted separate analyses for the 5 discrete domains of the ASQ-3. Two-sided *P* values <.05 were considered significant other than for the 5 ASQ-3 domain-specific analyses, in which a threshold of 2-sided *P* < .01 was used to correct for multiple comparisons.

To assess the sensitivity of our findings to selection and attrition bias, we descriptively compared the baseline characteristics of those included with those not included in the analysis. We performed propensity score adjustment to our primary analysis (model 2), in which propensity scores reflected the likelihood of being in the exposed vs the unexposed group given the covariates and the probability of missing outcome data. This score was calculated among all participants for whom exposure status was known (ie, those who completed all criteria but the ASQ-3), and it was calculated as Pr(E = 1|X) × Pr(O|X,E), in which Pr is the probability; E is the exposure status, and E = 1 if infected; O indicates whether outcome data were available vs missing; and X is the vector of baseline covariates. We also tested for associations between the exposure and outcome under extreme scenarios (an extreme case analysis) in which all children missing the outcome data were assumed to be neurodevelopmentally delayed or not delayed.

Last, we completed a sensitivity analysis excluding participants who were exposed, who were detected by serology alone without a prior negative antibody test, to account for uncertainty regarding the precise timing of incident infection. Analyses were conducted with R, version 4.3.2 (R Project for Statistical Computing)^35^ and Stata/BE, version 18.0 (StataCorp LLC)^36^ software.

## Results

**Figure 1.**
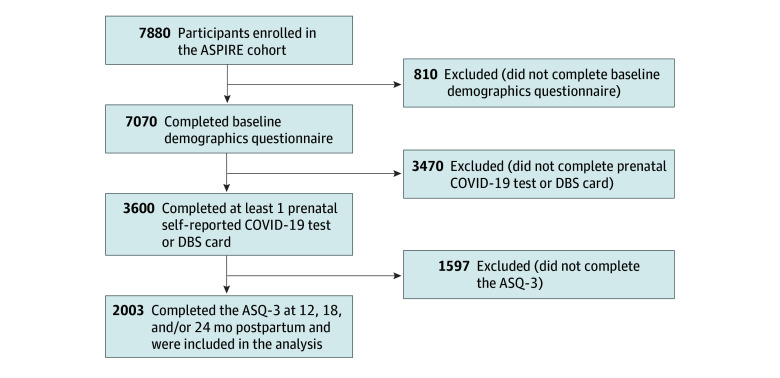
Flowchart of the ASPIRE (Assessing the Safety of Pregnancy in the Coronavirus Pandemic) Trial Population Selection ASQ-3 indicates Ages & Stages Questionnaire, Third Edition; DBS, dried blood spot.

A total of 7880 pregnant individuals from all 50 states and Puerto Rico initiated study activities (Figure 1). Complete information to assess exposure and outcome status refined those eligible for inclusion to 2003 participants (mean [SD] age, 33.3 [4.2] years) who enrolled before 10 weeks’ gestation and completed study activities (Figure 1). A total of 1469 of these participants (73.3%) were recruited via the BabyCenter platform, and the remaining 534 (26.7%) were recruited via the Society for Assisted Reproductive Technology. Among this cohort, 91 participants (4.5%) were Asian, 39 (1.9%) were Black, 169 (8.4%) were Hispanic, 1759 (87.8%) were White, and 75 (3.7%) were multiracial or other race and ethnicity; 1750 (87.4%) had earned a college degree.

Among the 2003 participants included in the study, exposure data in the form of both self-report and surveillance DBS cards were completed by 708 participants (35.3%), 799 (39.9%) submitted self-report exposure data only, and 496 (24.8%) submitted DBS cards but not self-reported exposure data. The ASQ-3 outcome data were submitted at all 3 time points (12, 18, and 24 months’ post partum) by the majority of participants (1192 [59.5%]).

A total of 217 of the 2003 participants (10.8%) experienced SARS-CoV-2 infection during pregnancy (exposed). Of the 217 participants who were infected, 163 (75.1%) were ascertained by self-report, 71 (32.7%) were detected by surveillance serology, and 17 (7.8%) were confirmed by simultaneous sources. Over half (122 [56.2%]) of maternal infections occurred during the first trimester of pregnancy. Most infections (189 [87.1%]) were primary infections in unvaccinated individuals (Table 1).

**Table 1.** Participant Characteristics by In Utero COVID-19 Exposure

Characteristic	Participant group^a^		
	Overall cohort (N = 2003)	Without prenatal infection (n = 1786)	With prenatal infection (n = 217)
Maternal age, mean (SD), y^b^	33.3 (4.2)	33.5 (4.2)	32.5 (4.2)
Race^c^			
Asian	91 (4.5)	83 (4.6)	8 (3.7)
Black	39 (1.9)	34 (1.9)	5 (2.3)
White	1759 (87.8)	1568 (87.8)	191 (88.0)
Multiracial or other^d^	75 (3.7)	73 (4.1)	2 (0.9)
Hispanic ethnicity^e^			
No	1789 (89.3)	1603 (89.8)	186 (85.7)
Yes	169 (8.4)	143 (8.0)	26 (12.0)
Educational level^f^			
<Bachelor’s degree	245 (12.2)	213 (11.9)	32 (14.7)
Bachelor’s degree	669 (33.4)	588 (32.9)	81 (37.3)
Graduate degree	1081 (54)	978 (54.8)	103 (47.5)
Yearly household income, $^g^			
<50 000	145 (7.2)	123 (6.9)	22 (10.1)
50 000-99 999	508 (25.4)	433 (24.2)	75 (34.6)
100 000-250 000	1047 (52.3)	950 (53.2)	97 (44.7)
>250 000	295 (14.7)	273 (15.3)	22 (10.1)
GAD-7 score^h^			
None to minimal	1287 (64.3)	1149 (64.3)	138 (63.6)
Mild to severe	682 (34.0)	608 (34)	74 (34.1)
PHQ-9 score^i^			
None to minimal	1055 (52.7)	944 (52.9)	111 (51.2)
Mild to severe	901 (45.0)	805 (45.1)	96 (44.2)
Time from enrollment to vaccine availability, mean (SD), wk^j^	17.9 (13.0)	18.4 (13.1)	14.5 (11.7)
Preterm birth (<37 wk’ gestation)^k^			
No	1848 (92.3)	1649 (92.3)	199 (91.7)
Yes	81 (4.0)	69 (3.9)	12 (5.5)
Infant sex^l^			
Female	854 (42.6)	766 (42.9)	88 (40.6)
Male	875 (43.7)	774 (43.3)	101 (46.5)
Female and male	14 (0.7)	10 (0.6)	4 (1.8)
Trimester of first SARS-CoV-2 infection^m^			
First	NA	NA	122 (56.2)
Second	NA	NA	42 (19.4)
Third	NA	NA	53 (24.4)
Breakthrough infection^n^			
No	NA	NA	189 (87.1)
Yes	NA	NA	28 (12.9)
COVID-19–related fever (>38 °C)			
No	NA	NA	177 (81.6)
Yes	NA	NA	40 (18.4)

Abbreviations: GAD-7, Generalized Anxiety Disorder 7^33^ (scores range from 0 to 21 points, with higher scores indicating a severe level of anxiety); NA, not applicable; PHQ-9, Patient Health Questionnaire score^34^ (scores range from 0 to 27, with higher scores indicating the presence of severe depressive symptoms).

^a^ Data are presented as No. (%) of participants unless indicated otherwise.

^b^ Data were missing for 238 participants.

^c^ Data were missing for 39 participants.

^d^ Multiracial includes participants who identified as any combination of Asian, Black, Native American, Pacific Islander, or White; other includes those who identified with groups not explicitly listed.

^e^ Data were missing for 45 participants.

^f^ Data were missing for 8 participants.

^g^ Data were missing for 8 participants.

^h^ Data were missing for 34 participants.

^i^ Data were missing for 47 participants.

^j^ COVID-19 vaccine availability began on April 19, 2021, for all adults in the US, including Puerto Rico and other territories.

^k^ Data were missing for 74 participants.

^l^ Data were missing for 260 participants.

^m^ The first infection is noted because 33 individuals experienced more than 1 SARS-CoV-2 infection within a pregnancy.

^n^ Maternal SARS-CoV-2 infection following COVID-19 vaccination.

The prevalence of abnormal ASQ-3 scores among 1757 children at age 12 months was 64 of 198 (32.3%) among those exposed vs 458 of 1559 (29.4%) among those unexposed (*P* = .39); among 1522 children at age 18 months was 36 of 161 (22.4%) among those exposed vs 279 of 1361 (20.5%) among those unexposed (*P* = .58); and among 1523 children at age 24 months was 29 of 151 (19.2%) among those exposed vs 230 of 1372 (16.8%) among those unexposed (*P* = .45) (eFigure in Supplement 1). The intraclass correlation coefficient for ASQ-3 scores, or correlation among observations within individuals, was 0.48 (95% CI, 0.42-0.55).

**Figure 2.**
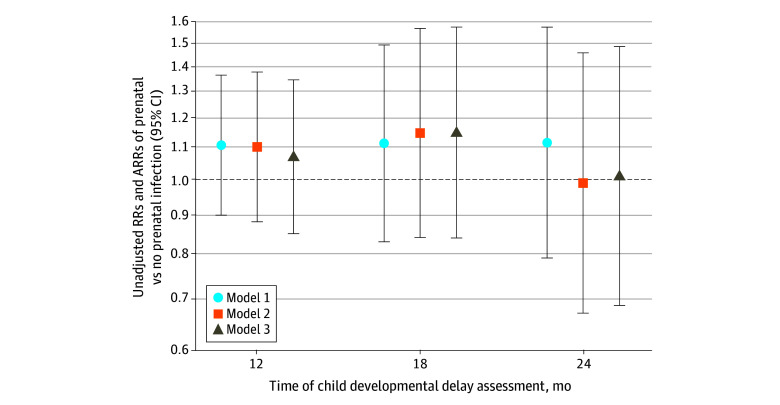
Association of Prenatal Infection vs No Prenatal Infection With Time of Developmental Delay Assessment by Model Model 1: unadjusted mixed-effects model including only the month of the Ages & Stages Questionnaire, Third Edition measurement; infection status; a month-by-infection interaction term; and random intercepts for participants. Model 2: adjusted for covariates measured at baseline (chosen a priori, as shown in Table 1) including maternal age, race, ethnicity, educational level, household income, mild to severe general anxiety (based on the Generalized Anxiety Disorder 7^33^), mild to severe depression (based on the Patient Health Questionnaire^34^), and the duration of time between enrollment and the time at which COVID-19 vaccines became available to all adults (April 19, 2021). Model 3: adjusted for covariates measured at baseline and infant sex and preterm birth (delivered <37 weeks’ gestation). Whiskers correspond to 95% CI ranges. ARRs indicates adjusted risk ratios.

After adjustment for confounders, the estimated percentage with abnormal neurodevelopment remained similar by exposure status (age 12 months, 32.2% [95% CI, 25.5%-38.9%] exposed vs 29.3% [95% CI, 27.0%-31.5%] unexposed; age 18 months, 23.2% [95% CI, 16.4%-30.0%] exposed vs 20.2% [95% CI, 18.0%-22.4%] unexposed; and age 24 months, 16.8% [95% CI, 10.6%-22.9%] exposed vs 16.9% [95% CI, 14.9%-19.0%] unexposed). There was no association between infection and abnormal ASQ-3 scores among those at age 12 months (ARR, 1.10 [95% CI, 0.88-1.38]), age 18 months (ARR, 1.15 [95% CI, 0.84-1.57]), or age 24 months (ARR, 0.99 [95% CI, 0.67-1.46]) (model 2 in Table 2). The addition of preterm delivery and infant sex did not change the conclusions at age 12 months (ARR, 1.07 [95% CI, 0.85-1.34]), age 18 months (ARR, 1.15 [95% CI, 0.84-1.57]), or age 24 months (ARR, 1.01 [95% CI, 0.69-1.48]) (model 3 in Table 2). Visual summaries of these primary findings are shown in Figure 2.

**Table 2.** Risk of Child’s Abnormal Developmental Screen at Ages 12, 18, and 24 Months by In Utero COVID-19 Exposure

Covariate	Risk ratio (95% CI)	Adjusted risk ratio (95% CI)	
	Model 1^a^	Model 2^b^	Model 3^c^
Time of child developmental delay assessment, mo^d^			
12	1.11 (0.90-1.36)	1.10 (0.88-1.38)	1.07 (0.85-1.34)
18	1.11 (0.83-1.49)	1.15 (0.84-1.57)	1.15 (0.84-1.57)
24	1.12 (0.79-1.57)	0.99 (0.67-1.46)	1.01 (0.69-1.48)
Maternal age	NA	1.06 (1.03-1.10)	1.06 (1.02-1.09)
Maternal race^e^			
Asian	NA	1.29 (0.97-1.73)	1.36 (1.02-1.80)
Black	NA	1.38 (0.95-2.01)	1.30 (0.88-1.91)
Multiracial or other^f^	NA	0.97 (0.68-1.38)	1.05 (0.74-1.48)
Maternal Hispanic ethnicity^g^	NA	1.01 (0.79-1.30)	1.00 (0.78-1.28)
Educational level^h^			
College degree	NA	1.07 (0.85-1.34)	1.09 (0.88-1.37)
Graduate degree	NA	1.06 (0.84-1.33)	1.04 (0.83-1.31)
Yearly household income, $^i^			
50 000-99 999	NA	0.81 (0.64-1.04)	0.79 (0.62-1.00)
100 000-250 000	NA	0.69 (0.54-0.88)	0.70 (0.55-0.89)
>250 000	NA	0.62 (0.46-0.84)	0.64 (0.47-0.86)
Mild to severe GAD-7 score^j^	NA	1.23 (1.07-1.42)	1.25 (1.09-1.44)
Mild to severe PHQ-9 score^j^	NA	0.92 (0.80-1.05)	0.92 (0.79-1.05)
Enrollment to vaccine availability, wk^k^	NA	0.99 (0.98-1.00)	0.99 (0.98-1.00)
Preterm birth (<37 wk’ gestation)^l^	NA	NA	1.54 (1.19-1.99)
Female infant sex^m^	NA	NA	0.74 (0.65-0.85)

Abbreviations: GAD-7, Generalized Anxiety Disorder 7^33^ (scores range from 0 to 21 points, with higher scores indicating a severe level of anxiety); NA, not applicable; PHQ-9, Patient Health Questionnaire^34^ (scores range from 0 to 27, with higher scores indicating the presence of severe depressive symptoms).

^a^ Unadjusted mixed-effects model including only the month of the Ages & Stages Questionnaire, Third Edition measurement; infection status; a month-by-infection interaction term; and random intercepts for participants.

^b^ Adjusted for covariates measured at baseline (chosen a priori, as shown in Table 1) including maternal age, race, ethnicity, educational level, household income, mild to severe general anxiety (based on the GAD-7), mild to severe depression (based on the PHQ-9), and the duration of time between enrollment and the time at which COVID-19 vaccines became available to all adults (April 19, 2021).

^c^ Adjusted for covariates measured at baseline and infant sex and preterm birth (delivered <37 weeks’ gestation).

^d^ Data are for prenatal infection (no prenatal infection is the reference). Estimates are generated using marginal probabilities from models containing an interaction between month and infection status. Mean imputation was used for maternal age in models 2 and 3 for those missing age (238 [12%]). A missing infant sex category was included in model 3 for those missing sex (260 [13%]).

^e^ White is the reference.

^f^ Multiracial includes participants who identified as any combination of Asian, Black, Native American, Pacific Islander, or White; other includes those who identified with groups not explicitly listed.

^g^ Non-Hispanic is the reference.

^h^ No college degree is the reference.

^i^ Less than $50 000 is the reference.

^j^ None to minimal is the reference.

^k^ COVID-19 vaccine availability began on April 19, 2021, for all adults in the US, including Puerto Rico and other territories.

^l^ Not premature is the reference.

^m^ Male is the reference.

Given the dynamic nature of in utero development, we explored whether the timing of infection was associated with our results but found no differences in outcomes across trimesters (Table 3). Among the 217 prenatal COVID-19 exposures, we compared febrile (40 [18.4%]) with nonfebrile (177 [81.6%]) and found no difference in ASQ-3 outcomes (Table 3).

**Table 3.** Risk of Child’s Abnormal Developmental Screen at Ages 12, 18, and 24 Months by Trimester of Infection, Breakthrough Infection, and Febrile Infection^a^

Primary exposure	Risk ratio (95% CI)	
	Unadjusted	Adjusted
**Child developmental delay assessment at age 12 months**		
Trimester of prenatal infection^b^		
First	1.15 (0.88-1.50)	1.12 (0.83-1.50)
Second	1.21 (0.81-1.82)	1.12 (0.72-1.76)
Third	0.92 (0.58-1.44)	0.92 (0.56-1.49)
Breakthrough infection^c^	0.88 (0.46-1.68)	0.88 (0.43-1.79)
Febrile infection^d^	1.24 (0.77-1.98)	1.08 (0.60-1.92)
**Child developmental delay assessment at age 18 months**		
Trimester of prenatal infection^b^		
First	1.31 (0.93-1.85)	1.43 (0.99-2.08)
Second	0.97 (0.49-1.93)	1.04 (0.53-2.03)
Third	0.81 (0.41-1.60)	0.73 (0.35-1.52)
Breakthrough infection^c^	0.26 (0.04-1.66)	0.29 (0.04-1.97)
Febrile infection^d^	0.34 (0.09-1.31)	0.30 (0.09-1.02)
**Child developmental delay assessment at age 24 months**		
Trimester of prenatal infection^b^		
First	1.21 (0.78-1.87)	1.03 (0.61-1.75)
Second	1.48 (0.80-2.74)	1.44 (0.74-2.78)
Third	0.68 (0.30-1.56)	0.66 (0.28-1.55)
Breakthrough infection^c^	0.29 (0.04-1.86)	0.31 (0.05-1.82)
Febrile infection^d^	0.40 (0.11-1.48)	0.34 (0.07-1.65)

^a^ Stratified estimates were generated using marginal probabilities from the fully adjusted model (model 3) plus an interaction between exposure and month of delay.

^b^ No prenatal infection is the reference.

^c^ Maternal SARS-CoV-2 infection following COVID-19 vaccination. Infection not preceded by vaccination is the reference.

^d^ Nonfebrile infection (≤38 °C) is the reference.

Given the expected differences in host immune response, we evaluated whether the potential risk to offspring differed based on whether infection occurred without prior vaccination vs as a breakthrough infection following vaccination. Our findings were null, with no increased risk posed by infections with or without prior vaccination (Table 3).

We conducted analyses to investigate whether risk might be limited to a specific ASQ-3 domain (eTable 1 in Supplement 1), with correction for multiple comparisons using a *P* < .01 significance threshold. We observed an increased risk of child abnormal problem-solving skills at age 12 months (RR, 1.57 [95% CI, 1.01-2.46]), which remained consistent after adjustment for confounders but with a wider CI (ARR, 1.56 [95% CI, 0.94-2.57]), and it was no longer observed at age 18 or 24 months.

Regarding missing data, baseline characteristics of those included vs those not included in the analysis are described (eTable 2 in Supplement 1). Our primary findings remained after propensity score adjustment (eTable 3 in Supplement 1) and extreme assumptions about missing data. Last, 42 of the 217 participants who were exposed were detected by positive surveillance serology alone without a prior negative serology test. We conducted a sensitivity analysis excluding these 42 participants to account for the possibility of an enduring serologic response from an asymptomatic infection preceding pregnancy, and the findings remained unchanged.

## Discussion

In this prospective cohort study of pregnant individuals and offspring, in utero exposure to maternal SARS-CoV-2 infection was not associated with abnormal neurodevelopmental screening scores of children through age 24 months. These findings are critical considering the novelty of the SARS-CoV-2 virus to the human species, the global scale of the initial COVID-19 outbreak, the now-endemic nature of the virus indicating ongoing relevance for pregnant individuals, the profound host immune response noted in many patients with COVID-19, and the accumulating evidence revealing sensitivity of the developing fetal brain to maternal immune activation.

Pandemics offer cautionary tales for potential clinical ramifications of maternal infection on subsequent generations. Notably, vertical transmission is not a prerequisite for adverse effects, allowing maternal COVID-19 (which, like influenza, generally does not cross the placenta) to wield a potential negative impact. The destructive mechanisms are attributed instead to maternal immune activation,^12,13,14^ which could influence placental and fetal immune and inflammatory signaling pathways.^13^ Indeed, maternal COVID-19, even remote from delivery, has been associated with elevations of cord blood cytokines, including interleukin (IL)-6 and IL-8,^37^ and enhanced production of interferon-γ, tumor necrosis factor, and IL-17 by neonatal immune cells.^38^ Animal studies suggest a mechanistic link among IL-17,^39^ IL-6,^40^ and other critical immune pathways as putative disruptors of in utero neurodevelopment.^18^ Further work is needed to understand potential fetal immune-protective mechanisms of perinatal infections and maternal immune activation.^41^

Recently, concern regarding neurodevelopment following in utero exposure to maternal COVID-19 was raised by a 2022 study of 7772 infants from a Massachusetts health system.^25^ Using electronic health record *International Statistical Classification of Diseases and Related Health Problems, Tenth Revision *codes, the authors identified an increase of 1.9 in adjusted odds (95% CI, 1.0-3.4; *P* = .04) of neurodevelopmental disorder diagnoses in the first 12 months of life among exposed infants.^25^ A follow-up study of 18 335 children by the same group clarified that the increased risk was restricted to male children,^26^ and while a trend persisted at age 18 months, the association was no longer statistically significant.^26^ The differences in results may relate to their use of billing code data, in which the basis for diagnosis and assessor are unknown and likely variable. In the present study, all offspring were assessed by maternal individuals with the ASQ-3, a systematic screening tool. Other differences between study populations could also explain discordant findings.

Relative advantages of the ASQ-3 outcome ascertainment include the granularity of assessment and ability to access individuals outside of the health care system. To date, 4 publications have examined whether fetal exposure to COVID-19 associates with altered ASQ-3 scores compared with contemporaneous controls.^19,20,21,22^ This literature is limited by small sample sizes (such as 18 infants),^22^ a short duration of follow-up (3 months,^21^ 6 months^20^), and a lack of adjustment for confounders.^19^ With this in mind, no differences in ASQ-3 scores were identified among 3 of the 4 studies^19,20,21^; in contrast, the fourth study with 18 infants observed a reduction in fine-motor scores among 9 exposed 8- to 10-month-olds compared with 9 unexposed infants (median, 49 [IQR, 45-55] vs 56 [IQR, 50-60]; *P* = .03).^22^ Our study may further clarify this burgeoning literature and add clinical reassurance for affected families.

While the scientific consensus resists a link between in utero COVID-19 exposure and impaired offspring neurodevelopment, the question remains whether societal responses to the pandemic impacted developmental trajectories. Certain studies comparing infants from a pandemic cohort with historic controls have raised concerns about lower ASQ-3 scores among children living during the pandemic.^20,42^ Critically, socioeconomic factors influence vulnerability, not only to infection itself but also regarding the ability to deploy resources in times of stress (eg, school closures) to mitigate sources of developmental harm. Our data support this theory, with the observed independent protective association of increasing household income with childhood ASQ-3 scores. Additional research is warranted to clarify the potential impact of societal measures on early development and the differential impact of these measures on different communities.

### Strengths and Limitations

The strengths of this study include its prospective nature, large scale, geographic diversity, early gestational enrollment, granularity of data, surveillance screening for asymptomatic infection, and duration of childhood follow-up, which is, to our knowledge, among the longest to date. Our recruitment strategy enabled us to uniquely capture exposures occurring during the first trimester, which is a period of explosive development and vulnerability. The granular nature of our data facilitated supplemental analyses to differentiate the timing of prenatal infection and other important features, such as whether fever accompanied an infection and whether vaccination preceded infection. Finally, our adjusted analyses yielded expected associations between covariates and outcome (eg, between preterm delivery and abnormal score), and the detection of more abnormal scores among male offspring may speak to the predictive performance of the ASQ-3 screening tool given the established sex difference in neurodevelopmental disorders.^43^ Finally, the ASQ-3 has established validity, sensitivity, and specificity and is widely used in research and clinical settings, allowing comparisons across cohorts.^44^

This study also has several limitations, including its volunteer recruitment using partnerships with an online fertility organization and BabyCenter, which restricts the distribution of baseline sociodemographic characteristics and hence generalizability to more vulnerable populations; imperfect retention and completion of study activities, which might impose selection bias; maternal completion of the ASQ-3 as a screening tool not to be misinterpreted as diagnostic; a serologic approach, which was launched before the availability of antigen testing; cohort size that, while larger than many studies, had relatively limited infections, particularly when examined by trimester; and recruitment design that was not powered to detect a prespecified difference in developmental delay. Not all participants returned their DBS cards, which might underestimate the incidence of asymptomatic infection. However, serologic data were available for over half of the participants. The pragmatic design rendered perfect monitoring for asymptomatic infection impossible, and the rollout of the COVID-19 vaccine in the middle of our study excluded subsequent detection of infection by serology. Regarding missing data, propensity score extreme case analyses provided further reassurance; however, caution is warranted when generalizing findings to broader populations.

## Conclusions

The data in this cohort study suggest that maternal COVID-19 was not associated with impaired neurodevelopment of offspring up to age 24 months. Ongoing, long-term studies across diverse cohorts are necessary to clarify the full spectrum of sequelae that may have resulted from the COVID-19 pandemic.

## Notes

Supplement 1. **eFigure.** Frequency of Child’s Abnormal Development Screen at 12, 18, and 24 Months, by In Utero COVID-19 Exposure **eTable 1.** Risk of Child’s Abnormal Developmental Screen at 12, 18, and 24 Months, by ASQ-3 Domain **eTable 2.** Baseline Characteristics by Inclusion Status **eTable 3.** Adjusted Risk of Delay Including Propensity Scores for Missing Outcome Data

Supplement 2.
Data Sharing Statement
