# Supplementary material for: In Utero Exposure to Maternal COVID-19 and Offspring Neurodevelopment Through Age 24 Months
Source: JAMA Netw Open. 2024 Oct 16;7(10):e2439792. doi: 10.1001/jamanetworkopen.2024.39792 (PMC11581627; doi:10.1001/jamanetworkopen.2024.39792)
Supplement: Supplement 1. — eFigure. Frequency of Child’s Abnormal Development Screen at 12, 18, and 24 Months, by In Utero COVID-19 Exposure eTable 1. Risk of Child’s Abnormal Developmental Screen at 12, 18, and 24 Months, by ASQ-3 Domain eTable 2. Baseline Characteristics by Inclusion Status eTable 3. Adjusted Risk of Delay Including Propensity Scores for Missing Outcome Data [file jamanetwopen-e2439792-s001.pdf]

Supplemental Online Content

Jaswa EG, Huddleston HG, Lindquist KJ, et al. In utero exposure to maternal COVID-19 and offspring neurodevelopment through age 24 months. *JAMA Netw Open*. 2024;7(10):e2439792. doi:10.1001/jamanetworkopen.2024.39792

eFigure. Frequency of Child's Abnormal Development Screen at 12, 18, and 24 Months, by In Utero COVID-19 Exposure
eTable 1. Risk of Child's Abnormal Developmental Screen at 12, 18, and 24 Months, by ASQ-3 Domain
eTable 2. Baseline Characteristics by Inclusion Status
eTable 3. Adjusted Risk of Delay Including Propensity Scores for Missing Outcome Data

This supplemental material has been provided by the authors to give readers additional information about their work.

© 2024 Jaswa EG et al. *JAMA Network Open*.

eFigure. Frequency of Child's Abnormal Developmental Screen at 12, 18, and 24 Months, by In Utero COVID-19 Exposure

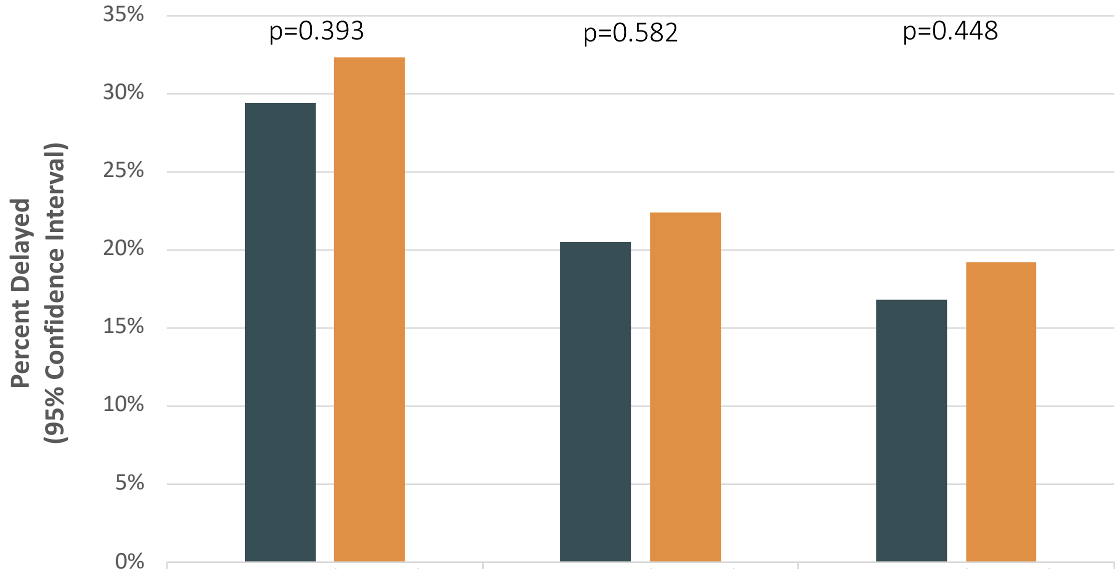

	12-month (N=1757)	18-month (N=1522)	24-month (N=1523)
No prenatal infection	29.4%	20.5%	16.8%
Prenatal infection	32.3%	22.4%	19.2%

P values are by chi-squared tests.

© 2024 Jaswa EG et al. *JAMA Network Open*.

eTable 1. Risk of Child's Abnormal Developmental Screen at 12, 18, and 24 Months, by ASQ-3 Domain

Month and ASQ-3 domain	Delay Among Unexposed No. (%)	Delay Among Exposed No. (%)	Unadjusted RR (99% CI)	Adjusted RR (99% CI)
Prenatal infection (ref: no prenatal infection): *12 months*[a,b]				
Communication skills	54 (3.5)	11 (5.6)	1.65 (0.71-3.87)	1.21 (0.43-3.44)
Gross motor skills	235 (15.1)	33 (16.7)	1.09 (0.75-1.59)	1.12 (0.74-1.69)
Fine motor skills	79 (5.1)	17 (8.6)	1.82 (0.95-3.50)	1.52 (0.73-3.20)
Problem solving skills	157 (10.1)	31 (15.7)	1.57 (1.01-2.46)	1.56 (0.94-2.57)
Social skills	186 (11.9)	29 (14.7)	1.23 (0.79-1.92)	1.00 (0.59-1.70)
Prenatal infection (ref: no prenatal infection): *18 months*[a,c]				
Communication skills	46 (3.4)	3 (1.9)	0.67 (0.16-2.72)	0.75 (0.20-2.76)
Gross motor skills	84 (6.2)	9 (5.6)	0.90 (0.40-2.05)	0.87 (0.36-2.13)
Fine motor skills	71 (5.2)	10 (6.2)	1.29 (0.56-2.96)	1.23 (0.51-2.96)
Problem solving skills	183 (13.5)	25 (15.5)	1.14 (0.70-1.86)	1.22 (0.72-2.08)
Social skills	47 (3.5)	5 (3.1)	0.94 (0.33-2.68)	0.89 (0.26-3.04)
Prenatal infection (ref: no prenatal infection): *24 months* [a,d]				
Communication skills	107 (7.8)	10 (6.6)	0.87 (0.38-2.00)	0.71 (0.27-1.82)
Gross motor skills	80 (5.8)	9 (6.0)	0.91 (0.39-2.13)	0.91 (0.37-2.24)
Fine motor skills	73 (5.3)	7 (4.6)	0.92 (0.36-2.32)	0.76 (0.25-2.36)
Problem solving skills	59 (4.3)	6 (4.0)	0.90 (0.32-2.54)	0.77 (0.22-2.69)
Social skills	78 (5.7)	13 (8.6)	1.52 (0.71-3.23)	1.25 (0.54-2.90)

RR: risk ratio; CI: confidence interval.

[a] Estimates are generated using marginal probabilities from models containing an interaction between month and infection status. Mean imputation was used for maternal age in the adjusted model for those missing age data (12%). A missing infant sex category was for those missing infant sex data (13%).

[b] ASQ data was available at 12 months for N=1559 unexposed and N=198 exposed participants.

[c] ASQ data was available at 18 months for N=1361 unexposed and N=161 exposed participants.

[d] ASQ data was available at 24 months for N=1372 unexposed and N=151 exposed participants.

© 2024 Jaswa EG et al. *JAMA Network Open*.

eTable 2. Baseline Characteristics by Inclusion Status

Baseline Characteristic	Excluded (N=5067)	Included (N=2003)
	Patients, No. (%)	
**Maternal age, mean (SD), y[a]**	32.7 (4.6)	33.3 (4.2)
**Race[b]**		
Asian	217 (4.3)	91 (4.5)
Black	226 (4.5)	39 (1.9)
White	3846 (75.9)	1759 (87.8)
Multiracial/Other[c]	275 (5.4)	75 (3.7)
**Hispanic ethnicity[d]**		
No	3987 (78.7)	1789 (89.3)
Yes	501 (9.9)	169 (8.4)
**Education[e]**		
Less than bachelor’s degree	1536 (30.3)	669 (33.4)
Bachelor's degree	1964 (38.8)	1081 (54.0)
Graduate degree	1162 (22.9)	245 (12.2)
**Household income[f]**		
<$50,000	698 (13.8)	145 (7.2)
$50,000-$99,000	1215 (24.0)	508 (25.4)
$100,000-$250,000	2079 (41.0)	1047 (52.3)
>$250,000	667 (13.2)	295 (14.7)
**General anxiety (GAD-7)[g]**		
None-Minimal	2528 (49.9)	1287 (64.3)
Mild-Severe	1871 (36.9)	682 (34.0)
**Depression (PHQ-9)[h]**		
None-Minimal	2064 (40.7)	1055 (52.7)
Mild-Severe	2317 (45.7)	901 (45.0)
**Enrollment to vaccine availability, mean (SD), w[i]**	15.9 (12.3)	17.9 (13.0)

a Age data was missing for N=1250 participants.
b Race data by self-report; data was missing for N=503 participants.
c "Multiracial" race includes those who identified as some combination of Asian, Black, Native American, Pacific Islander, and White; “other” race indicates those who identified with groups not explicitly listed.
d Hispanic ethnicity data by self-report; data was missing for N=579 participants.
e Education data was missing for N=405 participants.
f Income data was missing for N=408 participants.
g GAD-7 refers to the General Anxiety Disorder-7 measure. Data was missing for N=668 participants.
h PHQ-9 refers to the Patient Health Questionnaire-9 measure. Data was missing for N=686 participants.
I Weeks between study enrollment and April 19, 2021, when the COVID-19 vaccine became available to all adults in the United States, including Puerto Rico and other territories. Data was missing for N=6 participants.

© 2024 Jaswa EG et al. *JAMA Network Open*.

eTable 3. Adjusted Risk of Delay Including Propensity Scores for Missing Outcome Data

Covariate	Adjusted RR (95% CI)
Prenatal infection (ref: no prenatal infection): *12 months*[a]	
Complete data (from Table 2)	1.07 (0.85-1.34)
Adjusted for propensity scores[b]	1.02 (0.81-1.30)
Prenatal infection (ref: no prenatal infection): *18 months*[a]	
Complete data (from Table 2)	1.15 (0.84-1.57)
Adjusted for propensity scores[b]	1.09 (0.79-1.51)
Prenatal infection (ref: no prenatal infection): 24 *months*[a]	
Complete data (from Table 2)	1.01 (0.69-1.48)
Adjusted for propensity scores[b]	0.96 (0.64-1.42)

RR: risk ratio; CI: confidence interval.

[a]Estimates are generated using marginal probabilities from models containing an interaction between month and infection status.

[b] Propensity scores are calculated as Pr(E=1 | X) * Pr(O | X,E) where E is the exposure status and E=1 if infected, O indicates whether outcome data is available, X is the vector of baseline covariates.

© 2024 Jaswa EG et al. *JAMA Network Open*.
