# Supplementary material for: In Utero Exposure to Maternal COVID-19 and Offspring Neurodevelopment Through Age 24 Months
Source: JAMA Netw Open. 2024 Oct 16;7(10):e2439792. doi: 10.1001/jamanetworkopen.2024.39792 (PMC11581627; doi:10.1001/jamanetworkopen.2024.39792)
Supplement: Supplement 2. — Data Sharing Statement [file jamanetwopen-e2439792-s002.pdf]

Data Sharing Statement

Jaswa. In Utero Exposure to Maternal COVID-19 and Offspring Neurodevelopment Through Age 24 Months. *JAMA Netw Open*. Published October 16, 2024.
doi:10.1001/jamanetworkopen.2024.39792

Data
Data available: Yes
Data types: Deidentified participant data, Data dictionary
How to access data: eleni.jaswa@ucsf.edu
When available: beginning date: 07-01-2029

Supporting Documents
Document types: None

Additional Information
Who can access the data: Researchers whose proposed use of the data has been approved
Types of analyses: For a pre-approved purpose
Mechanisms of data availability: After approval of a proposal with a signed data access agreement
